# Giant Cell Arteritis Causing Isolated Pulmonary Artery Aneurysm

**DOI:** 10.1016/j.jaccas.2025.106235

**Published:** 2025-12-11

**Authors:** Ivka R. Stimach, Adil F. Vural, Marissa Edmiston, Ahmed Elghawy, Celeste Santos Martins, Mohamed Farhan Nasser

**Affiliations:** a Department of Internal Medicine, MetroHealth Medical Center/Case Western Reserve University, Cleveland, Ohio, USA; b Department of Rheumatology, Cleveland Clinic Foundation, Cleveland, Ohio, USA; c Heart and Vascular Center, MetroHealth Medical Center/Case Western Reserve University, Cleveland, Ohio, USA; d Department of Arthritis & Rheumatology, Mount Sinai Medical Center, Miami Beach, Florida, USA; e Pathology & Laboratory Medicine Institute, Cleveland Clinic Foundation, Cleveland, Ohio, USA

**Keywords:** giant cell arteritis, pulmonary artery aneurysm

## Abstract

**Background:**

Pulmonary artery aneurysm (PAA) is considered a rare finding, with an estimated incidence of 1 in 14,000.

**Case Summary:**

We present a case of PAA caused by giant cell arteritis isolated to the pulmonary artery, in which surgical intervention followed by clinical monitoring was pursued over immunosuppressive therapy.

**Discussion:**

The etiology of PAAs is variable, with reported cases found to be secondary to malignancy, infection, congenital diseases, autoimmune diseases, and vasculitis. Of the reported cases for vasculitis, one of the rarer etiologies includes giant cell arteritis, an autoimmune vasculitis involving large and medium-sized vessels such as the temporal artery and aorta, and the great vessels.

**Take-Home Messages:**

This case highlights a rare vasculitic etiology of pulmonary artery aneurysm formation. Prompt recognition and surgical intervention may prevent serious complications, especially in aneurysms at unusual sites.

## Graphical Abstract

**Figure:**
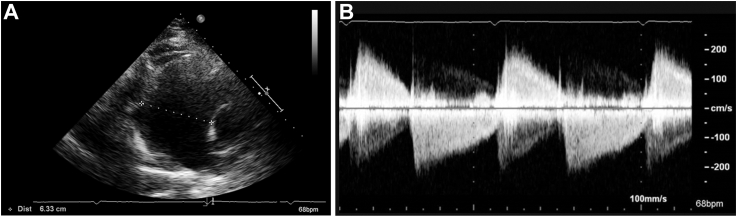

## History of Present Illness

An 82-year-old woman was seen by her primary care doctor after a trip to Florida, after which she noticed worsening fatigue and shortness of breath, which she attributed to a prior COVID infection. She denied cardiac symptoms such as chest pain and leg swelling. She also denied any headaches, jaw claudication, scalp tenderness, or vision changes. Review of systems was negative for fever, weight loss, night sweats, rash, or lymphadenopathy.

**Take Home Messages**

• This case highlights a rare vasculitic etiology of pulmonary artery aneurysm formation, with only a few reported cases in the literature.
• Prompt recognition and surgical intervention may prevent serious complications, especially in aneurysms at unusual sites.

## Past Medical History

The patient's medical history was significant for ulcerative colitis, hyperlipidemia, type 2 diabetes mellitus, and hypertension. She had no smoking history. Her family history was significant for coronary artery disease in her father, requiring coronary bypass surgery in his 60s, and aortic aneurysm in her mother, who underwent surgery in her late 60s.

## Differential Diagnosis

Initial differential diagnoses, based on symptoms and transthoracic echocardiography (TTE) findings of an enlarged pulmonary artery and severe pulmonary regurgitation, included pulmonary hypertension, anomalous pulmonary artery, coronary fistula, carcinoid syndrome, infectious microorganisms, and inflammatory diseases such as Takayasu arteritis.

## Investigations

**Figure 1:**
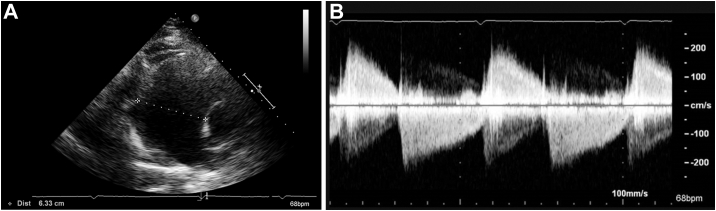
Findings on Transthoracic Echocardiography (A) Image reveals a severely dilated pulmonary artery measuring 6.3 cm. (B) Continuous-wave Doppler shows steep deceleration across the pulmonic valve, consistent with severe pulmonary regurgitation.

**Figure 2:**
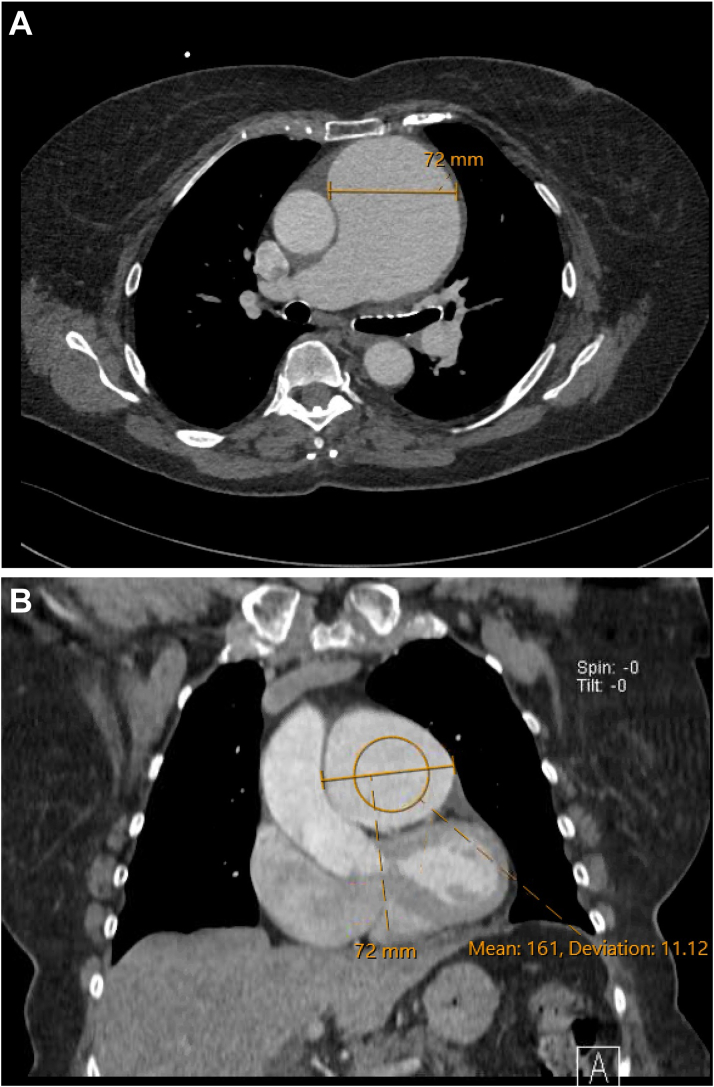
Findings on Contrast-Enhanced Chest Computed Tomography (A) Axial image shows the pulmonary artery aneurysm measuring 7.2 cm. (B) Coronal image shows the pulmonary artery aneurysm measuring 7.2 cm.

During the patient's initial outpatient appointment, TTE findings from 4 years prior were reviewed that noted mild pulmonic valve regurgitation with a dilated pulmonary artery of 4.9 cm in diameter. Based on these findings, as well as new symptoms of exertional dyspnea, a new TTE was performed, and showed a pulmonary artery aneurysm (PAA) measuring 6.3 cm (Figure 1A, Video 1), a right ventricular systolic pressure of 48 mm Hg, consistent with pulmonary hypertension along with a normal right ventricular function, and severe pulmonary regurgitation (Figure 1B, Video 2). Transesophageal echocardiography confirmed severe pulmonary regurgitation and a PAA with a diameter of 6.8 cm (Video 3). Contrast-enhanced chest computed tomography revealed the PAA, measuring up to 7.2 cm in diameter (Figures 2A and 2B). The remaining large vessels, imaged with computed tomography angiography of the neck, chest, and abdomen, did not show any aneurysm, stenosis, or wall thickening. Right heart catheterization revealed a right atrial mean pressure of 10 mm Hg, right ventricular pressure of 45/10 mm Hg, pulmonary artery pressure of 40/20 mm Hg (mean: 27 mm Hg), and a pulmonary capillary wedge pressure of 14 mm Hg. The cardiac index and cardiac output measured by thermodilution were 2.9 L/min/m^2^ and 5.2 L/min, respectively. Despite imaging findings, initial laboratory evaluation was largely unremarkable, with normal liver and kidney function and normal inflammatory markers.

**Figure 3:**
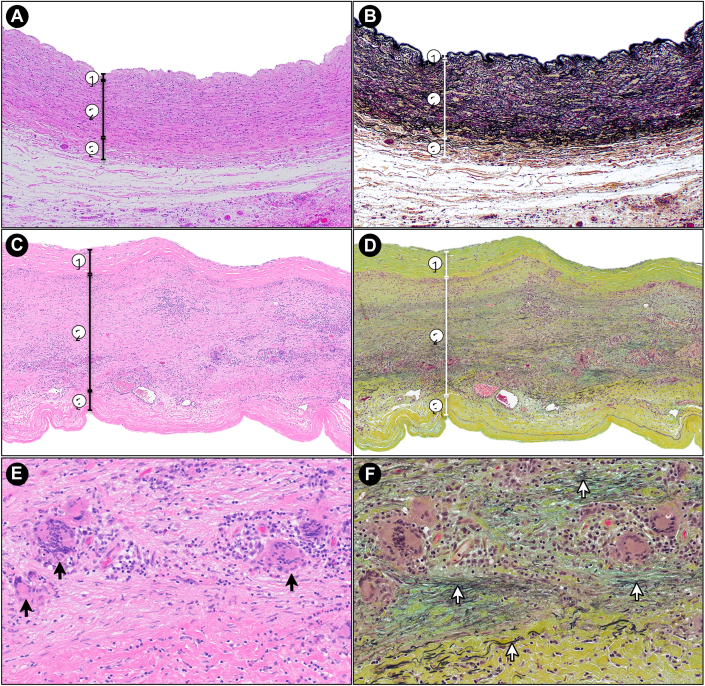
Histologic Findings of the Pulmonary Artery (A, C, and E) Hematoxylin and eosin staining. (B, D, and F) Movat pentachrome staining, highlighting elastic fibers in black and fibrosis in green-yellow. (A and B) Normal pulmonary artery histology. The three layers are clearly identified: (1) tunica intima, (2) tunica media, and (3) tunica adventitia. In the Movat-stained section (B), note the abundant elastic lamellae within the tunica media (2), highlighted in black. (C to D) Low-magnification views of the patient's pulmonary artery. The tunica media (2) is expanded by a dense inflammatory infiltrate with associated destruction of elastic fibers and fibrosis. (E and F) High-magnification views of C and D. Numerous multinucleated giant cells (black arrows in E) are seen within the inflammatory infiltrate in the tunica media. The corresponding Movat stain (F) demonstrates near-complete loss of elastic fibers, with only occasional fragmented remnants identified (white arrows).

The patient was diagnosed with giant cell arteritis (GCA) limited to the main pulmonary artery, without evidence of aortic or cranial involvement. Histological examination showed an exuberant inflammatory process in the media with numerous large multinucleated giant cells and associated lymphoplasmacytic infiltrates. Multinucleated giant cells extended into the adventitia. Movat stain demonstrated marked elastic fiber fragmentation and loss, and medial thinning without significant adventitial fibrosis. Special stains (Grocott-Gömöri Methenamine Silver, Periodic Acid–Schiff, and Fite) were negative for micro-organisms (Figure 3).

## Management

Given the enlarging PAA with associated symptoms, the patient underwent pulmonary artery replacement with pulmonic valve repair. Owing to surgical removal of the affected vascular segment, along with absence of cranial manifestations, and normalization of inflammatory markers, a decision was made to pursue clinical monitoring with serial imaging rather than initiate immunosuppressive therapy.

## Outcome and Follow-Up

At follow-up, the patient reported continued improvement in dyspnea and remained free of aortic and cranial symptoms. Repeat contrast-enhanced chest computed tomography demonstrated an intact pulmonary artery graft, with no residual disease or new aneurysmal changes. Inflammatory markers were within normal limits.

## Discussion

The estimated incidence of PAA is 1 in 14,000.^1^ Although various etiologies of PAA have been described in the literature, they are broadly categorized into congenital, acquired, and idiopathic groups, each encompassing a wide spectrum of underlying conditions. Congenital causes typically include structural cardiac anomalies such as patent ductus arteriosus, ventricular or atrial septal defects, and connective tissue disorders such as Ehlers-Danlos syndrome and Marfan syndrome. Acquired etiologies encompass infectious diseases (eg, syphilis, tuberculosis, fungal infections), vasculitis, pulmonary arterial hypertension (PAH), neoplasms, and iatrogenic factors such as prior cardiac surgery, thoracic instrumentation (eg, chest tubes), and conventional angiography.^5^

Among these categories, congenital causes represent the most common etiology of PAA. Within the vasculitis subgroup, pulmonary artery aneurysm formation due to GCA, as seen in our patient, is exceedingly rare, with few cases reported in the literature.^2^, ^3^, ^4^^,^^6^^,^^7^ A 2020 review of PAAs identified 46 cases attributed to autoimmune vasculitis, with only a single case associated with GCA.^8^ Of those with an underlying vasculitis process, on average aneurysmal size ranged from 8 to 10 cm in diameter and almost always involved distal pulmonary circulation.^8^

Understanding the underlying etiology of PAA is essential not only for guiding treatment, but also in identifying patients that may be at increased risk for aneurysm rupture or dissection. PAA rupture and dissection are exceedingly rare and, when detected, carry a >50% mortality rate.^9^ In patients with PAA without PAH, dissection can occur in up to 19% of patients, and the pulmonary artery trunk is the most common site involved. Only 15% of these dissections were diagnosed prior to their death.^5^ Of the 48 cases of PAA dissection documented since 1862, 43 cases were reported postmortem, with only 5 documented clinically.^10^ In the low-pressure pulmonary system, progressive increase in vessel wall tension from hemodynamic forces may accelerate aneurysm growth and increase the risk of rupture, as defined by Lapace's law.^3^^,^^9^, ^10^, ^11^ Careful assessment should focus on conditions that create persistent hemodynamic stress on the pulmonary vasculature as seen in PAH, intracardiac shunt flow, and valvular pathologies.^5^

The management strategy for PAA is dependent on the underlying etiology and presurgical risk. Conservative management includes targeted treatment toward the underlying etiology, combined with serial surveillance to monitor the size of the aneurysm. In the current case, a diagnosis of GCA was established postoperatively, as it could not be determined based on the available clinical presentation, imaging findings, or laboratory investigations. In such scenarios, the management approach involves radiological surveillance to monitor growth of the PAA, as well as surgical consultation when indicated.

Pulmonary vasodilators, which include endothelin receptor antagonists, phosphodiesterase inhibitors, prostaglandins, and calcium-channel medications, are often used in patients with PAH. Immunosuppressive therapy is used in cases of vasculitis associated with PAAs. Our case was reviewed with the operating surgeons, who confirmed that no additional grossly abnormal findings were identified intraoperatively for the rest of pulmonary trunk. No immunosuppressive therapy was used in our patient given the absence of clinical manifestations, the intraoperative findings, and the normalization of inflammatory markers after her surgery. This presentation resembles clinically isolated aortitis, in which patients may demonstrate thoracic aortic and branch vessel involvement despite having normal inflammatory markers.^12^ In a matched-cohort study, patients with clinically isolated aortitis who largely did not receive immunosuppressive therapy showed no significant difference in the incidence of new aneurysms or complications.^13^ The decision to initiate immunosuppression in such cases should be individualized, taking into account imaging findings, particularly branch vessel involvement, the risk of complications, patient comorbidities, and the potential adverse effects of therapy. Ideally, this decision should be made through multidisciplinary discussion, including input from rheumatology.

Surgical therapy in PAAs is associated with high mortality and morbidity, especially in patients with PAH. Although there is no scientifically proven surgical threshold, it is suggested to operate on PAAs of >5.5 cm.^5^ Routine imaging surveillance should be obtained to monitor growth of the PAA. Surgical consultation should be strongly considered in the presence of symptoms, rapid growth of the aneurysm (≥0.5 cm/year), compression of adjacent structures, thrombus formation in the aneurysm, presence of PAH, and presence of rupture or dissection. Endovascular treatments including coils and vascular plugs have been used in iatrogenic causes and small-branch aneurysms.^5^^,^^8^ Although these endovascular techniques have been described in case reports, there is a lack of specific scientific criteria regarding their use.

## Conclusions

This case represents one of the few documented instances of PAA associated with GCA, underscoring its rarity. Patients may not present with the classic findings of GCA such as cranial manifestations and abnormal inflammatory markers. Prompt recognition and surgery may prevent serious complications, especially in aneurysms at unusual sites.

## Funding Support and Author Disclosures

The authors have reported that they have no relationships relevant to the contents of this paper to disclose.

## Appendix

Video 1 Transthoracic Echocardiography Showing the Pulmonary Artery Aneurysm

Video 2 Transthoracic Echocardiography: Color Doppler Across the Pulmonic Valve Showing Severe Pulmonary Regurgitation

Video 3 Transesophageal Echocardiography Showing the Pulmonary Artery Aneurysm
